# ACTIVITY DIVERSITY ASSOCIATED WITH INCOME LEVEL AMONG US ADULTS BUT LESS SO FOR RACIAL/ETHNIC MINORITIES

**DOI:** 10.1093/geroni/igae098.0345

**Published:** 2024-12-31

**Authors:** Alexis Santos-Lozada, Soomi Lee

**Affiliations:** The Pennsylvania State University, University Park, Pennsylvania, United States; The Pennsylvania State University, University Park, Pennsylvania, United States

## Abstract

Active lifestyle is a key to healthy aging. Emerging research has shown that individuals with greater activity diversity are happier and healthier. Activity diversity, defined as the breadth and evenness of activity engagement across different types, may also allow opportunities to build human capital. This study examined whether activity diversity is associated with family income and its interaction with race/ethnicity. Data come from adults (n=115,771; Mage=45.62; 51% female; 37% racial/ethnic minorities) who participated in the American Time Use Survey between 2011 and 2022, excluding for 2020 due to the COVID-19 pandemic. An activity diversity index was calculated as conceptualized by Blau, using responses to a time-use questionnaire that covered all activities in which the respondent participated during the previous day. Generalized linear models revealed higher income to be associated with higher activity diversity. Further, there were differences in family income and activity diversity by race/ethnicity. Compared to non-Hispanic whites, racial/ethnic minorities reported earning less and engaging in less diverse activities. Moreover, the association between activity diversity and family income was moderated by race/ethnicity, such that higher family income was not as predictive of activity diversity among racial/ethnic minorities as it was among non-Hispanic Whites. Findings suggest that activity diversity may not only indicate one’s lifestyle, but also relate to one’s resources and capacity to explore new things in their life. Less resources available for racial/ethnic minorities may limit their opportunity to engage in diverse activities thought to improve human capital.

